# Distribution and habitat preferences of the stag beetle *Lucanus cervus* (L.) in forested areas of Poland

**DOI:** 10.1038/s41598-020-57738-9

**Published:** 2020-01-23

**Authors:** Robert Kuźmiński, Artur Chrzanowski, Andrzej Mazur, Paweł Rutkowski, Dariusz J. Gwiazdowicz

**Affiliations:** 1 Faculty of Forestry, Department of Forest Entomology, Poznan University of Life Sciences, ul. Wojska Polskiego 71C, 60-625 Poznań, Poland; 2 Faculty of Forestry, Department of Forest Sites and Ecology, Poznan University of Life Sciences, Poznań, Poland; 3 Faculty of Forestry, Department of Forest Pathology, Poznan University of Life Sciences, Poznań, Poland

**Keywords:** Forest ecology, Forestry

## Abstract

The incorporation of *Lucanus cervus* (L.) in Annex 2 of the EU Habitats Directive imposed on EU member countries the obligation to prepare protection plans and undertake adequate measures aimed at the preservation of this species. A necessary precondition for their implementation was connected with the identification of current localities of *L. cervus*. This paper presents the distribution of *L. cervus* localities in Poland, identified on the basis of a survey conducted in the areas administered by the State Forests. Habitat preferences for the selection of biotopes by stag beetles were evaluated in terms of forest-site types. This will facilitate effective protection of *L. cervus* by indicating potential biotopes for this species, particularly in areas with high abundance. The survey showed the presence of *L. cervus* in 176 localities distributed in 47 forest districts. Most of them were found in three main areas comprising forests in the areas of Zielona Góra, Wrocław and the Świętokrzyskie Mts. They constitute large-scale refuges. In 98% of cases the development of *L. cervus* was associated with oaks *Quercus robur* and *Quercus petraea*. Other host plants included *Fagus sylvatica* and *Acer pseudoplatanus*. The age of trees colonised by stag beetles ranged from 70 to 248 years, 134 years on average. *Lucanus cervus* was most frequently found in deciduous mesic forest sites (41% of localities) and deciduous mixed mesic forest sites (24% of localities). Over 90% of recorded localities are situated in forested areas, understood to include stands, residual trees and stumps, as well as localities at forest edges and along roads in the vicinity of forests.

## Introduction

Public awareness and acceptance of the need for nature conservation and preservation of biodiversity are growing in individual countries. This is indicated by the implementation of the Natura 2000 programme in the EU, aimed at the preservation of the European natural heritage. Its assumptions and objectives are definitely justified; nevertheless, protection of valuable nature elements may prove to be difficult. While protection measures in relation to habitats and birds, or vertebrates more generally, are proving to be increasingly effective, we are facing much greater difficulties in the case of invertebrates. These problems are connected with the very small size of invertebrate specimens and limited public knowledge of how to recognise some invertebrate species, as well as insufficient information on the distribution of their localities. It is not difficult to identify large beetles such as *Lucanus cervus* (L.), since even schoolchildren have no problem identifying male specimens. For this reason this species may be used as a perfect example in educating the public and as an umbrella species^1,2^, in consequence of which the habitats of saproxylic beetles will be protected and thus biodiversity will be effectively preserved^3,4^.

The stag beetle is one of the most charismatic saproxylic beetles in Europe^5^. In Poland it is the only species from the genus *Lucanus* Scop. and it has been covered by legal protection since 1952^6^. However, due to its imposing size and characteristic impressive mandibles this insect may be easily identified and thus has become an iconic symbol of nature conservation; on the other hand, it is considered to be a valuable collectible specimen and unfortunately it is subject to illegal trade^7^. This in turn leads to the question whether, aiming at species protection, we should conduct surveys and prepare maps of localities, which would facilitate protection of their localities or, on the contrary, would rather lead to illegal exploitation of the species by collectors. According to data from 2005 the stag beetle ranked fourth among the most popular Internet purchase and sale offers for protected insect species^7^ from the Polish Red Data Book of Animals and the IUCN Red List of Threatened Species. These potential threats have frequently led to decisions in Poland not to publish information on new localities of rare insect species. However, such an approach also may not ensure their adequate protection. It may also result in unintended destruction of the biotope, e.g. in the course of forest maintenance interventions, which had not been preceded by adequate nature surveys. It is particularly important to remember that commercial forestry is considered to be the primary cause of the dramatic decline in the population size and extinction of a majority of the *L. cervus* population in the Czech Republic^1^. Since the primary criterion when establishing the threat to beetle species is connected with the evaluation of the degree of threat to their micro- or macrohabitats^8^, the authors of this study consider it necessary to present the current distribution of *L. cervus* localities in Poland in commercial forests administered by the State Forests National Forest Holding, which manages forests owned by the State Treasury, covering approx. 90% of the forested area of Poland. This will facilitate an update of the map of *L. cervus* distribution in Europe, but also indicate the most threatened, i.e. isolated, populations and enable the development of a comprehensive programme for the protection of this species. This is particularly important when effective protection of beetles is achieved solely by protection of their habitat^8^. Moreover, it should also be remembered that the status of the stag beetle as a critically endangered species (EN – endangered after the Polish Red Data Book of Animals, which corresponds to the A – E criteria according to IUCN) creates an urgent need to identify localities of this species in Europe and to assess its population size^3,4,9^, particularly since information on its distribution in Europe is far from complete^3^ or is obsolete. For this reason, studies have been conducted in many countries to identify localities of *L. cervus* and to develop adequate species-monitoring methods^1,4,5,10–22^.

The main aim of this paper is to supplement data on the number and distribution of stag beetle localities in Poland. Moreover, we would like to present characteristics of stag beetle biotopes in the context of forest-site types and indicate the main host plants for this insect in Poland. Additionally, the effect of economic activity of foresters on the forest environment and thus the number of localities of this species was analysed. Such knowledge would allow more effective protection of *L. cervus* by indicating potential biotopes for this species, particularly in areas of its greatest abundance. This study may also provide foundations for a system of economic measures that would be either admissible or prohibited in areas of stag beetle occurrence.

## Material and Methods

The study is based on data collected within the framework of a survey of NATURA 2000 sites and species organised and financed by the State Forests National Forest Holding in the years 2006–2007. The survey was connected with Poland’s accession the EU and implementation of legal regulation on species and habitat conservation. The survey covered all forest districts in Poland and was conducted and supervised by a group of specialists affiliated to Polish universities or conservation organizations. The body of data was submitted to the authors of this paper for corroboration and analysis. This information was verified and confirmed during field work in later years (up to 2018). The data were combined with the information contained in the “Bank Danych o Lasach” database^23^.

The work was conducted in two stages and covered the entire forested area of Poland administered by the State Forests (approx. 30% of the country area and 77% of the forested area), excluding national parks and nature reserves. National parks, occupying approx. 2% of the area of Poland, and nature reserves have other forms of ownership and management. Moreover, with scarce exceptions *L. cervus* has not been reported in those areas. In turn, forests administered by the State Forests National Forest Holding (comprising 430 forest districts) have not to date been covered by comprehensive studies to obtain information on the distribution of the stag beetle and its localities. For this reason it was decided to conduct the research, the results of which are presented in this paper.

The first stage of the research, carried out in 2006–2007 within the Natura 2000 national habitat and species survey, comprised preliminary identification of stag beetle localities and separation of such localities into the following categories:

- cemeteries (including also closed cemeteries with old tree specimens, administered by the State Forests, having an administrative forest address),
- stands (stands having an administrative forest address, accessible in the “Bank Danych o Lasach” database) including also stumps (included when observations were made on stumps left after felling trees in forest areas of the State Forests) and residual trees (included when the age of the tree, on which insect observation was recorded, exceeded the age of the surrounding stand),
- forest boundaries (including single trees growing at forest edges and in open areas),
- parks,
- roadsides (included trees forming avenues along roads of various categories),
- settlements (included trees growing at settlements belonging to the State Forests).

In the first stage of research, data were collected on any reports/observations concerning the stag beetle, including observations of imagoes, traces of their colonisation, and reports by State Forest employees and local residents. The latter reports may be sources of significant data for monitoring saproxylic insects^24^.

In the second stage, conducted in 2010–2018, data from the first stage of research were verified in the course of field studies performed by the authors. Localities were confirmed by monitoring the area indicated as a biotope of *L. cervus*. The presence of beetles was assessed during the period of their activity (from May to August), by remains (body fragments such as mandibles of male specimens, exoskeletons) of insects killed by natural predators (Corvidae, Chiroptera, Insectivora) and by corridor exits located in the ground in the vicinity of stumps. The potential presence of larvae or coccoliths at tree bases and stumps was not investigated because of the legal ban prohibiting the disturbance of biotopes of protected species. In view of the 3- to 4-year generation of *L. cervus*, some localities were checked several times in the course of the study.

In the case of the localities in which the presence of *L. cervus* was recorded, the following data were collected:

- the tree species on which living insects were observed,
- the category of the area.
  The following data for the stands were included:
- species composition, determined according to the criteria binding in the State Forests and recorded in the Forest Management Plans of individual forest districts (Plan Urządzania Lasu - PUL),
- the age of the main stand (according to PUL),
- the forest-site type according to PUL.

The age of single trees (residual trees, trees growing at settlements belonging to the State Forests, trees forming avenues along roads) was determined on the basis of records contained in forest management plans, prepared at 10-year intervals for all State Forests units.

In the case of trees where stag beetles were reported, their species and age were specified wherever possible. For the category of stands, as well as for several other justified cases, additional data concerning the forest-site type were assumed on the basis of information contained in the Bank Danych o Lasach^23^ database.

## Results

### Distribution of the species in Poland

**Figure 1:**
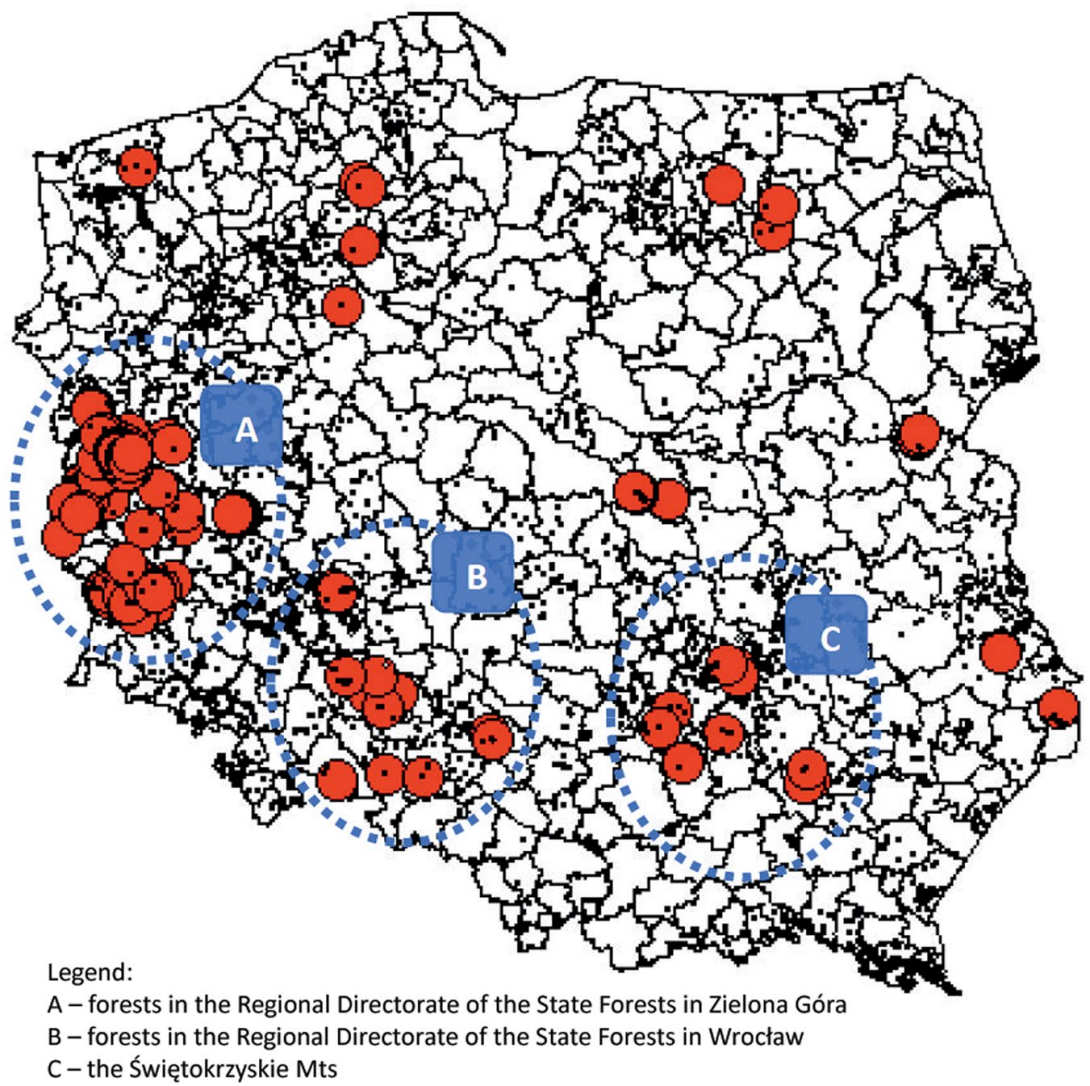
Map of Poland with forest district boundaries indicated, and showing localities of *Lucanus cervus* known from forest surveys in 2006–2007, with 176 localities confirmed.

**Figure 2:**
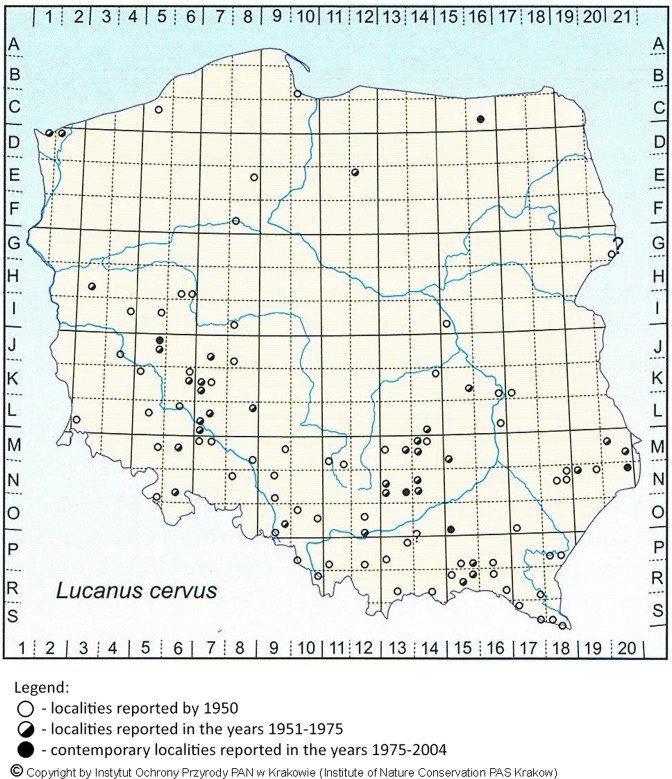
Localities of *Lucanus cervus* in Poland based on the Polish Red Book of Animals - Invertebrates^25^.

The survey provided 318 records ascribed to 176 localities, located in 47 forest districts (Appendix 1). Most of them are situated in three main clusters and about a dozen localities scattered throughout Poland (Fig. 1). In the three main areas of the species’ occurrence (forests of the Regional Directorate of the State Forests in Zielona Góra, forests in the area of Wrocław and the Świętokrzyskie Mts.) we can identify large-scale refuges. Other localities are strongly isolated, which indicates seriously threatened insect populations. Observing the results with historical data (Fig. 2) given in the Polish Red Book of Animals - Invertebrates^25^, it may be stated that the occurrence of *L. cervus* was confirmed in the main localities in which it is concentrated, as well as in northern, central and eastern Poland. No information was obtained on the presence of *L. cervus* in south-eastern Poland, despite the fact that data from the 1990s^26,27^ indicate relatively numerous populations in the localities of that region. This may have resulted from the limited coverage of the region by surveys because of its affiliation to other administrative units, including national parks. However, it should be stated here that the distribution of stag beetle localities in Poland until 1950 covered a more or less compact area, forming a belt extending from the area of Zielona Góra towards Wrocław and further in the southern part of Poland towards its eastern boundary. At present there are three main separated areas in this region, representing isolated clusters, which may confirm that in Poland its occurrence range is rather disjunctive, with a marked trend towards gradual decline in the number of localities^25,27^. In future this may lead to permanent isolation of local populations and depletion of the species gene pool^28,29^.

The map shown in Fig. 1 gives the current distribution of stag beetle localities in Poland. On the basis of this it will be possible to identify the most isolated, and thus the most threatened, populations of the species, as well as provide more effective protection. It also addresses the proposals concerning the urgent need to identify *L. cervus* localities in Europe^4^.

### Host species

The age of trees with recorded signs of stag beetle activity (e.g. exit holes of beetles at stump roots) ranged from 70 to 248 years (134 years on average). These results confirm earlier observations^26^ indicating that this species prefers oak stands aged 80–130 years. Analysis showed that 51% of *L. cervus* localities were found in stands of that age range. A comparable share, i.e. as many as 46% of localities, was in older stands aged 132–248 years, while the fewest localities (3%) were reported in the youngest stands of 70–75 years old. This observation is of great importance, as it provides evidence that the presence of trees with a minimum age of 70 years is a condition for the maintenance of continuous development of this species.

### Habitat preferences

**Figure 3:**
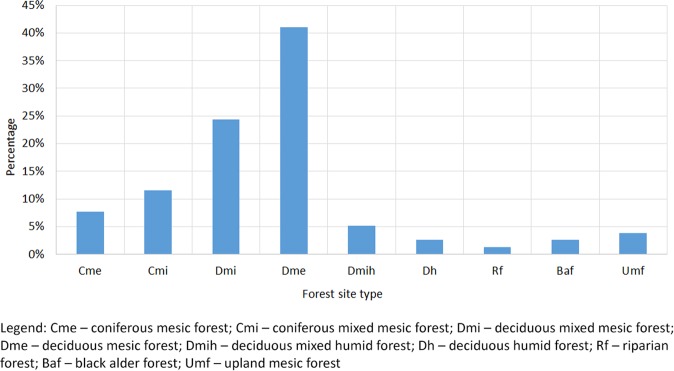
The relationship between *Lucanus cervus* localities and specific forest-site types.

A lack of adequate monitoring methods makes it difficult to characterise habitats of the stag beetle. As a result, descriptions of habitats for the species are scarce^5,20,30^. In order to identify relationships of *L. cervus* with commercial forest types, localities where the species occurred were associated with commercial forests classified in Poland according to the forest site classification, taking into consideration the species composition of the stand, the species composition of the vegetation cover, and soil conditions based on assessment of fertility and soil moisture content. Analysis showed that 77% of localities were associated with three main forest-site types (Fig. 3). The largest number of stag beetle localities (41%) was reported in fresh deciduous mesic forest, characterised by a deciduous stand, associated mainly with two plant community types: oak, hornbeam-oak or lime-hornbeam-oak forests *Querco-Carpinetum* (in Poland covering the geographical variants *Stellario-Carpinetum*, *Galio sylvatici-Carpinetum* and *Tilio-Carpinetum*), and beech community *Melico uniflorae-Fagetum* (also classified as *Galio odorati-Fagetum* or *Asperulo*-Fagetum), mainly found on loess soils, brown and rusty brown soils. The primary species in the vegetation cover, distinguishing deciduous mesic forest from other forest site types, includes *Lamiastrum galeobdolon* (=*Galeobdolon luteum*), *Carex sylvatica*, *Melica uniflora*, *Galium odoratum (=Asperula odorata), Dentaria bulbifera, Actaea spicata, Sanicula europaea and Pulmonaria obscura*. In forest-site types classified as deciduous mixed mesic forest, 24% of localities were found, while in forest sites classified as coniferous mixed mesic forest there were 12% of localities. The deciduous mixed mesic forest sites are formed mainly of mixed oak-pine or beech-pine forests, representing primarily the communities of *Calamagrostio arundinaceae-Quercetum petraeae*, *Fago-Quercetum, Luzulo pilosae-Fagetum* and less frequently *Potentillo albae-Quercetum petraeae*. Rusty soils are the dominant substrates in such forest-site types. The coniferous mixed mesic forest sites are generally *Querco roboris-Pinetum* communities, but frequently they are also substitute communities with dominance of pine, covering potential communities listed in the description for the deciduous mixed mesic forest-site type. Rusty soils are also frequently associated with coniferous mixed mesic forest, although typically in less fertile subtypes than in the case of deciduous mixed mesic forest.

### Localities of the species

**Figure 4:**
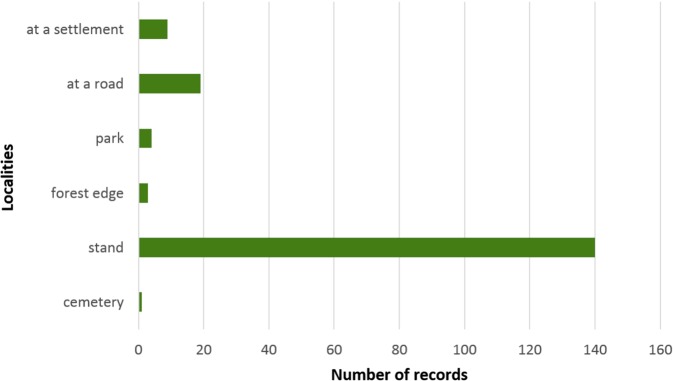
Biotopes of *Lucanus cervus*.

When evaluating the distribution of stag beetle localities in Poland it was found that 92% of them are directly associated with forested areas, covering areas administered by the State Forests. Localities situated outside forested areas are often in the nearvicinity of forests (Fig. 4).

Among the total number of localities identified, 176 were dense stands. The others were more open sites such as stumps, roadside avenues, and trees growing at forest edges outside forests (e.g. at forest settlements). Such a situation indicates that the species protection system for the stag beetle should definitely extend outside forest management per se and should also focus on areas not administered by the State Forests.

## Discussion

The stag beetle is one of the largest beetle species, not only in Poland but throughout Europe. It is saproxylophagous, i.e. a species that, in its development cycle, requires the presence of dead wood or dying trees. In the past it was found throughout almost all of Europe, whereas at present it has become scarce or extinct in many areas^6,31^. Its populations are currently thought to be either absent from, or has become extinct in at least 13 countries, while in many others (12) it is an endangered species^9^. For example, in the Pardubice region of the Czech Republic it is considered a critically endangered species^1^. It is assumed that in Europe the stag beetle population is declining because of a reduction in its typical habitats^9^, and depletion or fragmentation of habitats sustaining the beetles is believed to be their greatest threat^5^. A similar opinion has also been expressed by other authors^10^, who consider that, apart from habitat fragmentation, expansion of new orchard plantations (cherry trees) is among the main threats to the stag beetle. The primary causes for the decrease in stag beetle populations in individual Czech regions include forest management methods leading to changes in the species composition and distribution, as well as increased shading in forest stands^1^. As a consequence of the considerable reduction in the proportion of dead and dying trees observed in European forests the species has been classified as threatened with extinction^32^. In Poland *L. cervus* is considered as EN (Endangered), which indicates a species facing a threat of extinction because of its small population, fragmented island distribution, and/or dramatic rate of population decline (in terms of the population size and/or area)^25^.

The stag beetle has been covered by species protection in Poland since 1952. The need to protect the species within the EU has also been acknowledged. For this reason it was incorporated in Annex II of the EU Habitats Directive (Council Directive 92/43/EEC of 21 May 1992 on the conservation of natural habitats and of wild fauna and flora), which imposes the obligation to determine the status of the species, to monitor it and to establish special protected areas within the Natura 2000 network^33^. This has resulted in many European countries implementing measures aimed at determining the current distribution and status of this species as well as ensuring its protection.

### Distribution of the species in Poland

Currently available information on the occurrence of the stag beetle in Poland is obsolete and considered to be no longer reliable^6,32^. The data cover a period of more than 150 years^6^. Most historical localities have not been confirmed after 1950 (Fig. 2), while additionally some reports are considered dubious, imprecise or insufficiently documented^25^. Despite intensive efforts to find stag beetles within the Natura 2000 programme^33^, there was a reduced number of confirmed localities, which, it is assumed, may have resulted from actual shrinkage and depletion of local populations^26,32^. According to data from the survey reported here, in Poland there are about a dozen scattered localities for *L. cervus*, among which large stag beetle populations are found only in western Poland, while in the rest of the country the populations are strongly fragmented and isolated^6^. This situation has also been observed in national parks and nature reserves. Data on the localities for *L. cervus* in legally protected areas in Poland were first presented by Kaźmierczak^34^, who mentioned eight national parks and five nature reserves. These localities need mostly to be considered as historical and not confirmed by recent data. For example, in the Białowieski National Park *L. cervus* was last recorded in 1942^35,36^. Current data are scarce and concern only four nature reserves^37–39^, three newly established Natura 2000 areas, which protect stag beetle localities^39,40^, and one landscape park^26^.

Despite the lack of specific information on the size of local stag beetle populations in Poland, data provided by the survey indicate that most *L. cervus* localities recorded both before 1950 and in later years, reported in the Polish Red Book of Animals - Invertebrates^25^, have been preserved. This may suggest that current forest management practice has had no major effect on the depletion of biotopes of this species. This is a crucial finding since, according to Nieto and Alexander^41^, the species is threatened as a consequence of depleted dead wood resources and decreasing numbers of old trees (caused by a reduced mean age of stands) resulting from intensive and excessive forest exploitation, particularly in eastern Europe. Forest management has also been identified as a factor causing a rapid reduction in the range of the stag beetle in the Czech Republic, where its populations were considered to be the most stable in Europe^1^. The data presented suggest that forests in Poland in many places (Fig. 1) continue to provide refuge for this rare insect. This also does not fully confirm the assumption that only in the past forests were the primary habitats for the stag beetle, because they used to be considerably less dense than the present-day commercial forests^42^. While the main pressure on saproxylic species, including the stag beetle, is exerted by forest management operations through elimination of dead wood and stumps, as well as felling old trees in forests and parks^43^, this was not manifested in a considerable decrease in the number of *L. cervus* localities in forests of Poland. Since 1991, Polish law on forests has included regulations providing for hollowed or dying trees and rotting wood to be left in the forest, which would obviously promote the occurrence of this beetle. The rationale for leaving stumps of felled trees and dead wood in the forests is that, it is assumed, their presence sustains the beetle populations by providing a biotope for larval development^5,11,43,44^.

The data obtained may be used for the preparation of a detailed map of *L. cervus* localities in Poland and in Europe. An attempt to assess the distribution of the stag beetle in Europe has already been made^9^, however, but with very few localities recorded in Poland after 1970. Most of the localities shown were either reported before 1 January 1970 or were given no specific date and as such are obsolete data. Despite a hypothesis suggesting that a lack of new data on the occurrence of stag beetles in Poland and several other countries after 1970 may have been related to the period of political unrest^9^, in reality it resulted from less interest in this species in Poland and a form of protection based on not publicising information on its distribution.

### Host species

The stag beetle is trophically associated mainly with oaks, while it may also colonise beech, hornbeam, elm or fruit trees^25^. Larval development of the stag beetle is also observed on representatives of *Salix*, *Populus*, *Tilia*, *Aesculus*, *Prunus* and *Fraxinus* species^45^, as well as *Betula*, *Alnus*, *Castanea sativa*^46^, *Malus sylvestris*^35^ or even coniferous trees (*Pinus* and *Picea*)^8,27^ and date palms *Phoenix dactylifera* L.^47^. The latest information indicates that stag beetles may also develop on *Quercus rubra*^30^. According to Harvey *et al*.^8^, oaks predominate among host plants (over 50%), while the shares of the other species do not exceed 10% for each. Results from the survey reported here indicate that in Poland *L. cervus* is associated primarily with oaks (*Quercus robur* and *Q. petraea*), which accounted for 98% of host species. Only in two cases was development of stag beetles associated with beech trees (*Fagus sylvatica*) and in one case with sycamore (*Acer pseudoplatanus*).

### Habitat preferences

The stag beetle is a thermophilous species preferring natural open stands, mainly oak forests and oak-hornbeam forests^6^. It selects first of all localities with southern exposure and a warm microclimate^47^. The following habitats are considered as its potential habitats^41^ in Poland:

- 9160 – sub-Atlantic and central European oak forests and oak-hornbeam forests *Carpinion betuli*,
- 9170 – oak-hornbeam forests *Galio-Carpinetum* (expanded to include the proposed mixed lime-oak-hornbeam forest *Tilio-Carpinetum*),
- 9190 – old acidophilous oak forests with *Quercus robur* on sandy plains,
- 91F0 – riparian mixed deciduous forests with oak *Quercus robur, Ulmus laevis* and *Ulmus minor*, ash *Fraxinus excelsior* or *Fraxinus angustifolia*, growing along large rivers (*Ulmenion minoris*).

In Romania the beetle was reported from the following habitats^48^: 9130 *Asperulo-Fagetum* beech forests, 91Y0 Dacian oak-hornbeam forests, 91M0 Pannonian-Balkanic Turkey oak-sessile oak forest with sessile oak, pine and acacia groves with pine and locust and a mixture of 9130 *Asperulo-Fagetum* beech forests and 91Y0 Dacian oak-hornbeam forests. However, the largest number of *L. cervus* specimens was recorded in the habitat 9170 *Galio-Carpinetum* oak-hornbeam forests with oak and hornbeam.

As was shown above, the stag beetle in Poland is associated mainly with oaks, which was confirmed by its habitat preferences. Among these habitat types deciduous forests predominate, followed by deciduous mixed forests. However, it was shown that stag beetles were also attracted by residual oak trees of low silvicultural value, growing in coniferous mixed forests. In view of the longer life of oaks compared with that of pines accompanying them in that forest-site type, such a situation even predisposes oaks left after felling of pines to colonisation by *L. cervus*. Thus it seems that leaving single oak trees after felling the surrounding stand is an important element in the protection of stag beetles, since residual trees comprise the dominant group of their localities. Stag beetles reported on residual oak trees in coniferous mixed mesic forest may also be seen as evidence that foresters, both in the past and at present, acknowledged the importance of both the stag beetle and old oak specimens, which have not been removed within typical forest management operations.

### Localities for the species

In Europe *L. cervus* is associated primarily with mature oak stands in lowland and upland areas^49^, mainly up to the altitude of 1700 m a.s.l.^48^, while in Poland it is generally up to 300–400 m a.s.l. The stag beetle is considered to be a species strongly dependent on prevailing temperatures, which is explained by its absence in areas with warmer climates (southern Spain, Portugal and Italy) as well as with cooler climates (northern Great Britain and Sweden)^9,50^. However, its occurrence in Saudi Arabia, where it is the most important pest of *Phoenix dactylifera*, causing considerable damage and tree die-back, as well as its presence in Northern Africa^47^, indicates that temperature is not the only factor determining colonisation of a specific biotope by *L. cervus*.

Although it was believed in the past that it is a species of vast forested areas^51^, it may also be found in more open areas and in urbanised habitats, such as gardens, parks, orchards, roadside trees and avenues^42,45,52–59^, colonising not only stumps and decomposing wood, but also e.g. fence posts or railway-track sleepers^9^. This confirms observations^8^ that unique associations of beetles are sometimes found in unnatural objects, apparently devoid of any value to nature.

**Figure 5:**
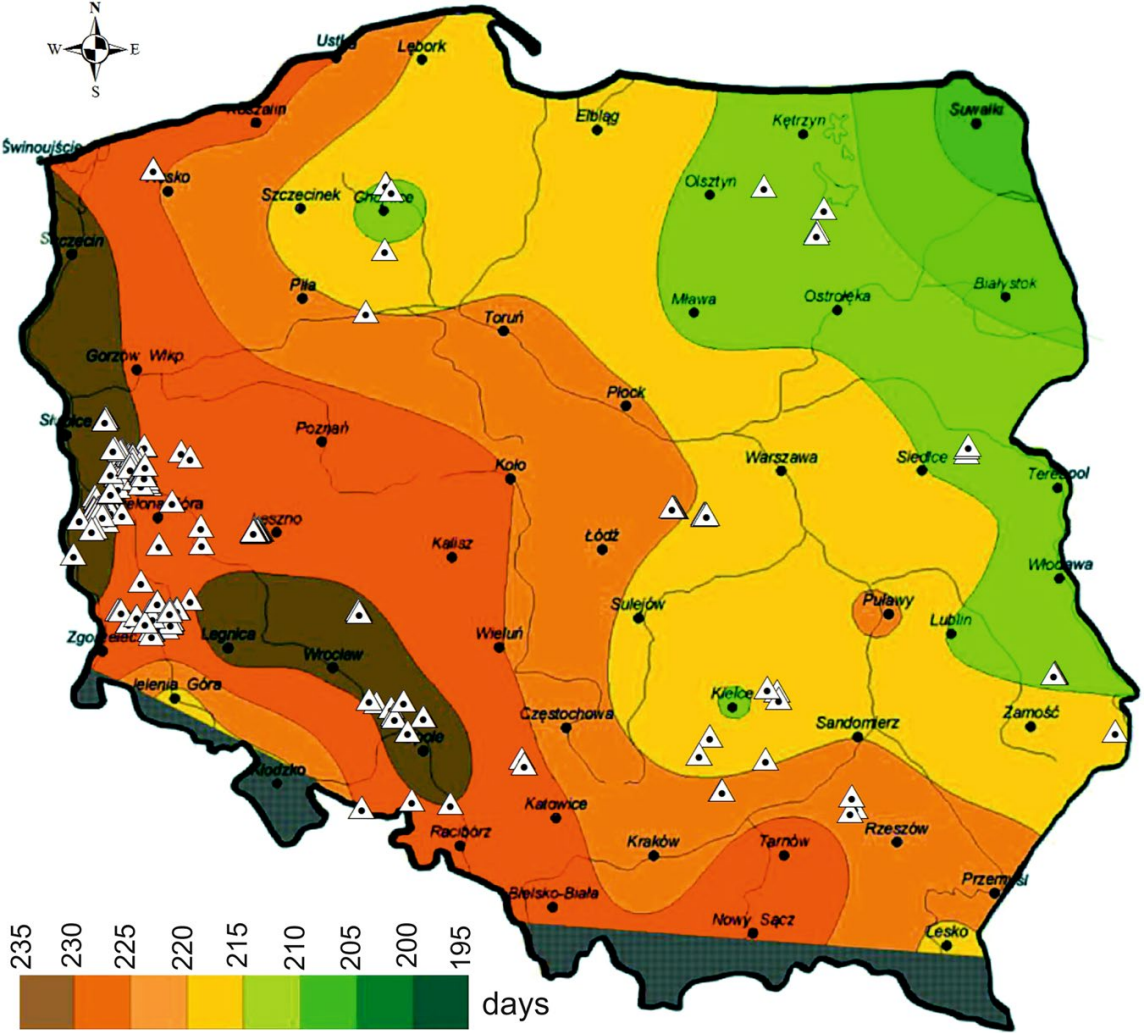
Distribution of *Lucanus cervus* localities (white triangles) (original) on a map showing duration of the meteorological vegetation growing period in Poland in the years 1981–2010^64^.

*Lucanus cervus* is often found, particularly in north-western Europe, in small forest biotopes either within or near cities^53,54,60^. In Great Britain stag beetles prefer the urban environment and they are found most frequently in gardens, as well as other urban areas, parks and hedgerows^9^. Such occurrences of *L. cervus* in urbanised areas were also reported in the Czech Republic, but not on such a large scale as in Great Britain or Belgium^1^. In Belgium, however, there is a marked differentiation of habitat preferences; in the Atlantic zone *L. cervus* is most often found in urbanised areas, while in the continental zone its most important habitats have been forests and forest edges^58^. The greater attractiveness of urban areas for stag beetles is most probably related to a warmer microclimate, which is reflected in a faster development rate of individual insect life-cycle phases^58^. It is also associated with less predator pressure and protection of host plants, since old trees are often preserved in parks, avenues, cemeteries, etc. It is assumed, however, that locations such as fence posts or railway-track sleepers are secondary localities and are rather substitutes, which probably may not ensure long-term occurrence of the beetles^1^. Outside Great Britain *L. cervus* is found primarily in forested areas^9^. On the European continent the largest number of localities has been recorded in oak stands, followed by parks, urban gardens and other urbanised habitats^9^. As a thermophilous species, in forests on heavy-loamy soils (in dense and shaded stands) many localities of stag beetles are found outside stands or at forest edges, while on sandy soils, in less dense and “warmer” stands with oaks, birches and pines, localities for this species are also found within stands^42^. Using the established model it was shown that in Italy forests were the most suitable habitats for *L. cervus*^43^, since they provide considerable amounts of large-sized wood debris^61,62^. In turn, the stag beetle has avoided areas covered with shrubs, arable land and developed areas^43^. The factor with most effect on the occurrence of the stag beetle was found to be altitude (a.s.l.), followed by the percentage of deciduous mixed forests^42^. Some role has also been played by temperature, the proportion of urbanised areas and soil type^58^. Although it was not the main aim of this paper, the occurrence of this species in Poland was compared depending on the length of the vegetation period (Fig. 5). This corresponds strictly to the effect of air temperature. This relationship will be investigated in the course of further research in the context of climate warming and resulting environmental changes.

Future research needs also to cover forests administered by other entities (e.g. private, church, community forests), as well as forests located in national parks, cities, towns and villages. Surveys should also include old roadside avenues, parks and cemeteries. Areas in the direct vicinity of human settlements also need to be surveyed, particularly since in Western Europe stag beetles have been observed in fragmented forest complexes and near cities^3,63^.

## Conclusions

1. While the stag beetle colonises diverse forest sites and several tree species, it was observed to prefer large forest complexes (refuges) with a marked proportion of oaks aged over 80 years.
2. In order to ensure its effective protection, focus should be on forest communities with a predominance of older oak trees, and forest management should have ecological emphasis and include such measures as leaving dying and rotting trees on site. Continuous monitoring is needed in such areas.
3. Temperature, which determines the length of the vegetation growth period, is a significant factor affecting distribution of *L. cervus* in Poland. This needs to be investigated further, with a view to identifying more precisely the factors determining the occurrence of this species, thus leading to its more effective conservation.

## Supplementary information

Supplementary information: Dataset 1.
