# Supplementary material for: Distribution and habitat preferences of the stag beetle Lucanus cervus (L.) in forested areas of Poland
Source: Sci Rep. 2020 Jan 23;10:1043. doi: 10.1038/s41598-020-57738-9 (PMC6978315; doi:10.1038/s41598-020-57738-9)
Supplement: Supplementary file 1 — Supplementary information: Dataset 1. [file 41598_2020_57738_MOESM1_ESM.doc]

Distribution and habitat preferences of the stag beetle *Lucanus cervus* (L.) in forested areas of Poland

Robert Kuźmiński, Artur Chrzanowski, Andrzej Mazur, Paweł Rutkowski & Dariusz J. Gwiazdowicz

**Appendix 1.** A list of *Lucanus cervus* localities with their locations given in the coordinate reference system (X, Y) in Poland

| **No.** | **Forest district** | **Locality** | **Species** | **Age of trees** | **GPS coordinates** | |
| --- | --- | --- | --- | --- | --- | --- |
|  |  |  |  |  | **X** | **Y** |
|  | Babimost | residual trees | Q | N | 274934,290 | 496440,697 |
|  | Bolesławiec | residual trees | Q | N | 254285,317 | 399975,868 |
|  | Bolesławiec | residual trees | Q | N | 255291,412 | 399884,404 |
|  | Bolesławiec | residual trees | Q | N | 255842,432 | 399540,837 |
|  | Bolesławiec | residual trees | Q | N | 255594,043 | 399645,964 |
|  | Bolesławiec | Stand | Q | 151 | 251854,768 | 396947,133 |
|  | Brzeg | forest edge | Q | N | 390489,243 | 344074,232 |
|  | Brzeg | Stand | Fs | 246 | 399425,426 | 341321,109 |
|  | Brzeg | Stand | Q | 126 | 401220,774 | 336855,429 |
|  | Brzeg | Stand | Q | 150 | 401226,759 | 335055,296 |
|  | Brzózka | at a settlement | Q | N | 223355,057 | 456823,906 |
|  | Brzózka | at a settlement | Q | N | 225926,015 | 462753,079 |
|  | Brzózka | at a road | Q | N | 225952,444 | 464014,424 |
|  | Brzózka | at a road | Q | N | 225857,950 | 464088,326 |
|  | Brzózka | at a road | Q | N | 225450,233 | 463072,188 |
|  | Brzózka | Stand | Q | 117 | 232652,580 | 461291,735 |
|  | Brzózka | Stand | Q | 129 | 226104,253 | 466975,532 |
|  | Brzózka | Stand | Q | 170 | 225851,100 | 463761,351 |
|  | Bytnica | at a settlement | Q | N | 244762,618 | 479493,745 |
|  | Bytnica | at a settlement | Q | N | 243628,767 | 492049,199 |
|  | Bytnica | at a road | Q | N | 244939,042 | 480583,386 |
|  | Bytnica | at a road | Q | N | 244766,459 | 487994,182 |
|  | Bytnica | at a road | Q | N | 245098,271 | 487345,559 |
|  | Bytnica | at a road | Q | N | 245132,778 | 487402,550 |
|  | Bytnica | at a road | Q | N | 245986,733 | 485601,598 |
|  | Bytnica | at a road | Q | N | 246092,908 | 486037,988 |
|  | Bytnica | at a road | Q | N | 246274,138 | 485497,840 |
|  | Bytnica | at a road | Q | N | 246373,801 | 484444,950 |
|  | Bytnica | at a road | Q | N | 247131,053 | 490997,629 |
|  | Bytnica | residual trees | Q | N | 240711,546 | 482665,107 |
|  | Bytnica | residual trees | Q | N | 238761,642 | 491532,100 |
|  | Bytnica | residual trees | Fs | 155 | 238009,756 | 489804,950 |
|  | Bytnica | park | ? | N | 245678,827 | 486501,754 |
|  | Chełm | stand | Q | 157 | 809144,172 | 362109,805 |
|  | Chmielnik | stumps | Q | 160 | 630842,397 | 309479,841 |
|  | Chocianów | residual trees | Q | N | 262820,296 | 396636,140 |
|  | Chocianów | residual trees | Q | N | 262772,459 | 396623,407 |
|  | Chocianów | residual trees | Q | N | 263303,473 | 395593,845 |
|  | Chocianów | residual trees | Q | N | 263302,706 | 395738,692 |
|  | Chocianów | residual trees | Q | N | 263154,976 | 395634,255 |
|  | Chocianów | residual trees | Q | N | 262960,293 | 393681,079 |
|  | Chocianów | residual trees | Q | N | 262818,927 | 393659,120 |
|  | Chocianów | forest edge | Q | N | 262927,014 | 395297,565 |
|  | Gryfice | stand | Qp | 136 | 252162,047 | 674730,911 |
|  | Gubin | residual trees | Q | N | 218202,217 | 460481,344 |
|  | Gubin | residual trees | Q | N | 220503,329 | 460024,074 |
|  | Gubin | residual trees | Q | N | 206229,729 | 457992,498 |
|  | Gubin | park | Q | N | 217043,809 | 470365,344 |
|  | Gubin | stand | Q | 89 | 215498,202 | 464698,619 |
|  | Gubin | stand | Qp | 158 | 215673,918 | 464386,166 |
|  | Gubin | stand | ? | ? | 215624,565 | 464362,488 |
|  | Gubin | stand | Qp | 228 | 218089,942 | 464544,966 |
|  | Gubin | stand | Qp | 248 | 217960,579 | 464302,758 |
|  | Gubin | stand | ? | ? | 217634,711 | 463215,746 |
|  | Gubin | cemetery | Q | N | 216242,735 | 472454,152 |
|  | Jędrzejów | stand | Q | 145 | 596581,857 | 323437,839 |
|  | Jędrzejów | stand | Q | 155 | 589776,287 | 312450,964 |
|  | Kaczory | stand | Q | 201 | 383791,752 | 585986,402 |
|  | Krosno | at a settlement | Q | N | 225678,657 | 486630,359 |
|  | Krosno | at a road | Q | N | 232213,629 | 485877,139 |
|  | Krosno | at a road | Q | N | 230002,642 | 484578,343 |
|  | Krosno | at a road | Q | N | 230684,138 | 484251,818 |
|  | Krosno | residual trees | Q | N | 229496,771 | 486204,134 |
|  | Krosno | residual trees | Q | N | 229931,889 | 484439,463 |
|  | Krosno | residual trees | Q | N | 230403,764 | 477497,751 |
|  | Krosno | stand | Q | 130 | 225969,977 | 474379,901 |
|  | Kup | residual trees | Q | N | 409811,132 | 326616,117 |
|  | Kup | park | Q | 129 | 419585,106 | 335724,638 |
|  | Kup | park | Q | N | 419392,854 | 335616,963 |
|  | Lubliniec | residual trees | Q | N | 480240,668 | 309647,037 |
|  | Lubliniec | residual trees | Q | N | 481569,713 | 306012,238 |
|  | Lubsko | residual trees | Q | N | 216116,463 | 452315,118 |
|  | Lubsko | stand | Qr | 126 | 213813,101 | 451664,666 |
|  | Lutówko | residual trees | Q | 126 | 395235,445 | 624968,652 |
|  | Maskulińskie | at a settlement | Q | N | 663067,921 | 636330,718 |
|  | Maskulińskie | residual trees | Q | N | 662785,701 | 634159,202 |
|  | Maskulińskie | residual trees | Q | N | 666898,491 | 650068,586 |
|  | Mielec | residual trees | Q | N | 683389,146 | 276581,456 |
|  | Mielec | stand | Q | 144 | 686319,194 | 279247,932 |
|  | Milicz | residual trees | Q | N | 379417,400 | 401844,996 |
|  | Milicz | residual trees | Q | N | 379327,226 | 401845,396 |
|  | Milicz | residual trees | Q | N | 379148,427 | 401591,354 |
|  | Milicz | forest edge | Q | N | 379724,910 | 400336,766 |
|  | Mircze | residual trees | ? | N | 847660,294 | 326693,309 |
|  | Namysłów | stand | Ap | 130 | 406916,737 | 345547,170 |
|  | Nowa Dęba (Buda Stalowska) | stand | Q | 122 | 684640,671 | 286607,734 |
|  | Nowa Sól | stand | Q | 132 | 255719,218 | 442523,351 |
|  | Oława | stand | Q | 121 | 385867,078 | 346452,314 |
|  | Oława | stand | Q | 111 | 385928,738 | 346324,658 |
|  | Ośno Lubuskie | stand | Qp | 105 | 222944,059 | 520480,755 |
|  | Ośno Lubuskie | stand | Qp | 134 | 222492,693 | 520973,257 |
|  | Ośno Lubuskie | stand | Qp | 134 | 222665,977 | 520653,188 |
|  | Pińczów | stumps | Q | N | 604261,784 | 289848,372 |
|  | Pińczów | stand | Q | 139 | 603903,898 | 290184,472 |
|  | Prószków | stand | Q | 136 | 412151,400 | 283869,964 |
|  | Prudnik | at a road | Q | N | 381159,875 | 279146,823 |
|  | Przemków | residual trees | Q | N | 267080,453 | 402503,854 |
|  | Przemków | residual trees | Q | N | 266743,670 | 402819,505 |
|  | Przemków | residual trees | Q | N | 266618,379 | 402678,736 |
|  | Przemków | residual trees | Q | N | 266570,419 | 402799,105 |
|  | Przemków | residual trees | Q | N | 266223,835 | 402810,469 |
|  | Przemków | residual trees | Q | N | 266866,686 | 402255,139 |
|  | Przemków | residual trees | Q | N | 266856,604 | 402196,319 |
|  | Przymuszewo (Osusznica) | residual trees | Q | 119 | 396234,006 | 665355,694 |
|  | Przymuszewo (Osusznica) | residual trees | Q | 119 | 395803,916 | 665372,666 |
|  | Przymuszewo (Osusznica) | residual trees | Q | N | 399197,728 | 661401,796 |
|  | Przytok | residual trees | Q | N | 263830,405 | 468576,765 |
|  | Radziwiłłów | stand | Q | 108 | 593006,781 | 461208,387 |
|  | Radziwiłłów | stand | Q | 70 | 594146,279 | 460989,428 |
|  | Radziwiłłów | stand | Q | 115 | 592864,171 | 460305,182 |
|  | Radziwiłłów | stand | Q | 75 | 594520,314 | 460396,956 |
|  | Ruszów | at a road | Q | N | 241551,240 | 397624,462 |
|  | Ruszów | residual trees | Q | N | 232136,471 | 401307,667 |
|  | Ruszów | stand | Q | 159 | 240110,233 | 395166,141 |
|  | Sarnaki | residual trees | Q | N | 755700,330 | 498745,995 |
|  | Sarnaki | residual trees | Q | N | 755419,833 | 501610,062 |
|  | Sarnaki | stand | Q | 80 | 756773,015 | 503386,297 |
|  | Skierniewice | residual trees | Q | N | 573768,999 | 465248,412 |
|  | Skierniewice | residual trees | Q | N | 573385,163 | 465209,482 |
|  | Skierniewice | stand | Q | 109 | 575227,795 | 464914,520 |
|  | Skierniewice | stand | Q | 109 | 574906,864 | 465076,019 |
|  | Skierniewice | stand | Q | 109 | 574906,279 | 464945,894 |
|  | Sława śląska | residual trees | Q | N | 281796,755 | 453693,094 |
|  | Strzelce Opolskie | at a road | Q | N | 436083,171 | 281926,233 |
|  | Suchedniów | at a settlement | Q | N | 639239,176 | 346978,808 |
|  | Suchedniów | residual trees | Q | N | 638196,760 | 351619,220 |
|  | Sulechów | residual trees | Q | N | 249611,434 | 479091,258 |
|  | Świebodzin | at a settlement | Q | N | 244542,190 | 495549,396 |
|  | Świebodzin | residual trees | Q | N | 246535,725 | 502934,025 |
|  | Świebodzin | residual trees | Q | N | 246558,815 | 503085,949 |
|  | Świebodzin | residual trees | Q | N | 239019,157 | 496040,209 |
|  | Świebodzin | residual trees | Q | N | 269468,532 | 499677,976 |
|  | Świętoszów | residual trees | Q | N | 249861,974 | 400993,125 |
|  | Świętoszów | residual trees | Q | 120 | 241872,525 | 398211,376 |
|  | Świętoszów | residual trees | Q | 90 | 251543,960 | 396987,173 |
|  | Świętoszów | residual trees | Q | 150 | 247018,984 | 394077,589 |
|  | Świętoszów | residual trees | Q | 150 | 246810,106 | 393914,501 |
|  | Świętoszów | residual trees | Q | 120 | 254737,738 | 407146,385 |
|  | Świętoszów | residual trees | Q | 110 | 254697,461 | 407016,200 |
|  | Świętoszów | residual trees | Q | 150 | 262469,664 | 402347,143 |
|  | Świętoszów | residual trees | Q | 130 | 262335,338 | 400472,127 |
|  | Torzym | at a road | Q | N | 228347,472 | 502379,964 |
|  | Torzym | residual trees | Q | N | 232790,253 | 501300,988 |
|  | Torzym | residual trees | Q | 100 | 237463,073 | 497000,035 |
|  | Torzym | residual trees | Q | N | 232410,761 | 500670,566 |
|  | Torzym | residual trees | Q | N | 231837,797 | 500434,295 |
|  | Torzym | residual trees | Q | N | 227115,344 | 501442,387 |
|  | Węgliniec | stand | Q | 179 | 251073,980 | 389649,530 |
|  | Węgliniec | stand | Q | 180 | 250677,631 | 389313,290 |
|  | Wipsowo | at a settlement | Q | N | 630041,859 | 663785,615 |
|  | Włoszakowice | stand | Q | 138 | 315899,179 | 451558,519 |
|  | Włoszakowice | stand | Q | 138 | 315811,181 | 451391,263 |
|  | Włoszakowice | stand | Qp | 138 | 315306,077 | 451599,509 |
|  | Włoszakowice | stand | Qp | 138 | 314990,620 | 451673,281 |
|  | Włoszakowice | stand | Qp | 138 | 314881,255 | 451687,684 |
|  | Włoszakowice | stand | Qp | 90 | 314751,348 | 451986,234 |
|  | Włoszakowice | stand | Qp | 138 | 314706,792 | 451707,614 |
|  | Włoszakowice | stand | Qp | 138 | 314641,496 | 451719,898 |
|  | Włoszakowice | stand | Qp | 128 | 316274,623 | 450798,522 |
|  | Włoszakowice | stand | Qp | 124 | 315726,893 | 450927,627 |
|  | Włoszakowice | stand | Qp | 124 | 315809,030 | 451008,956 |
|  | Włoszakowice | stand | Qp | 128 | 315983,064 | 450289,878 |
|  | Włoszakowice | stand | Qp | 124 | 315727,371 | 450355,955 |
|  | Włoszakowice | stand | Qp | 124 | 315481,545 | 450397,054 |
|  | Włoszakowice | stand | Qp | 124 | 315384,702 | 450451,837 |
|  | Włoszakowice | stand | Qp | 108 | 315069,286 | 450596,574 |
|  | Włoszakowice | stand | Qp | 108 | 315066,434 | 450349,189 |
|  | Włoszakowice | stand | Qp | 104 | 314744,728 | 450762,420 |
|  | Włoszakowice | stand | Qp | 104 | 314631,745 | 450680,432 |
|  | Włoszakowice | stand | Qp | 114 | 314452,534 | 450849,606 |
|  | Włoszakowice | stand | Qp | 148 | 316497,211 | 451545,486 |
|  | Włoszakowice | stand | Qp | 148 | 316571,706 | 451535,461 |
|  | Włoszakowice | stand | Qp | 148 | 316409,155 | 451343,018 |
|  | Włoszakowice | stand | Qp | 148 | 316117,043 | 451212,594 |
|  | Włoszakowice | stand | Qp | 128 | 317015,875 | 450836,793 |
|  | Żagań | residual trees | Q | N | 244543,229 | 419720,910 |

Ap – *Acer pseudoplatanus*

Fs – *Fagus sylvatica*

Qp – *Quercus petraea*

Qr – *Qurcus robur*

Q – *Qurcus robur* or *Q. petraea*

N – age not specified

? – no specific data for the locality
